# Classroom practice for understanding pointers using learning support system for visualizing memory image and target domain world

**DOI:** 10.1186/s41039-017-0058-4

**Published:** 2017-09-02

**Authors:** Koichi Yamashita, Ryota Fujioka, Satoru Kogure, Yasuhiro Noguchi, Tatsuhiro Konishi, Yukihiro Itoh

**Affiliations:** 1 Faculty of Business Administration, Tokoha University, 1230 Miyakoda, Kita-ku, Hamamatsu, Shizuoka 431-2102 Japan; 2 Graduate School of Informatics, Shizuoka University, 3-5-1 Johoku, Naka-ku, Hamamatsu, Shizuoka 432-8011 Japan; 3 Faculty of Informatics, Shizuoka University, 3-5-1 Johoku, Naka-ku, Hamamatsu, Shizuoka 432-8011 Japan; 4 Shizuoka University, 3-5-1 Johoku, Naka-ku, Hamamatsu, Shizuoka 432-8011 Japan

**Keywords:** Education for programming, Program visualization system, Domain world model, Classroom practice

## Abstract

Pointers are difficult learning targets for novice learners of C programming. For such difficult targets, introducing a system visualizing program behaviors is generally expected to support learners to understand the targets. However, visualization in existing systems often conceals the concrete value of variables such as pointers; the way in which each visualized object is located on the memory is not made explicit. In order to address this issue, we focused on a program visualization system called TEDViT. It visualizes simultaneously and synchronously the memory image that is the field that presents the concrete value of variables and the target domain world that is the field that presents logically the data structures processed by the program. We consider that observing and comparing program code, memory image, and target domain world with TEDViT could work for understanding pointers. TEDViT visualizes the status of the target domain world according to the visualization policy defined by the teacher in order to allow teachers to set their instruction content based on the growing variety of learner background knowledge. We also consider that this feature could support teachers’ instructions and class managements appropriately, and improving teachers’ performance by TEDViT’s support would bring improvement of learners’ understanding. We conducted classroom practice for understanding pointers in connection with a memory model, thus introducing TEDViT to a real class. Analysis of answered scores in a questionnaire conducted after the practice suggests that our practice using TEDViT provided useful supports for participants to understand pointers. It also suggests our practice had a certain effect to reduce uneven levels of understanding among participants. Based on these results, we describe that classroom practices in our framework could support learners to understand pointers and support teachers to manage the class.

## Introduction

As information technology has pervaded our society in recent years, improving the productivity of program codes has been expected increasingly. Many institutes of higher education have organized programming classes for various students. Teachers are required to teach learners with various levels of background knowledge in limited time and hence to achieve efficient and effective programming education.

Thus far, several program visualization systems have been developed to support novice learners in understanding program codes (Pears et al., 2007). These systems visualize the data structures processed by the target programs in certain ways and support learners in understanding the programs and algorithms by making their behavior visible. Sorva, Karavirta, and Malmi (2013) pointed out the following difficulties faced by novice programming learners:

- Novice learners often see particular programming concepts merely as pieces of code rather than as active components of a dynamic process that occurs at runtime.
- Novice learners often cannot grasp so-called notional machine which is an abstraction of the computer in the role of executor of programs.
- The runtime world of the notional machine and the domain world of target processed by program code are hidden and are not clearly visible in program code.
- Novice learners often fail to trace programs step by step because of insufficient understandings of statement sequencing and insufficient ability to keep track of program state.

Program visualization systems support novice learners to overcome these difficulties by visualizing the logical data structures processed by target programs. Introducing these systems to classes is expected to allow learners to cultivate a better understanding of program and hence to contribute efficient and effective programming education (Naps et al., 2002).

However, in some case depending on learning target, teachers who introduced an existing system into their classes sometimes feel unsatisfied about the visualizations provided by the system. The typical learning target is pointers, for which teachers tend not to accept system’s visualizations. Their visualizations of logical data structures have fixed abstraction level, and hence, these often conceal the concrete value of variables such as pointers; the way in which each visualized object is located on the memory is not made explicit. The behaviors of pointer processing such as operations on pointer variables and passing pointer arguments in function calls are difficult for novice learners to understand by observing the visualizations.

We consider this undesirable situation is caused because many of existing systems have focused only on visualizing logical data structures and have made light of visualizing statuses of the notional machine. An expert programmer might have an image of the status of a target domain world according to the memory image of the variables provided by a debugger or tracer and might grasp changes in the world status based on the program code. That is, perceiving successfully the statuses of the notional machine and the statuses of the target domain world, and the relationships between them would derive better understanding of the program code like an expert programmer. We consider that the failure of many of novice programmers to understand programs is caused by their poor ability to perceive the relationships among the program code, memory image, and status of the target domain world.

As suggested by the investigation results of Milne and Rowe (2002) and Lahtinen, Ala-Mutka, and Jävinen (2005), pointer is a learning target that many of novice learners find hard to understand. In order to support learners to understand pointers, we focused on a program visualization system called TEDViT (Kogure et al., 2014), which is an acronym for Teacher Explaining Design Visualization Tool, and conducted classroom practice for understanding pointers introducing TEDViT. It visualizes simultaneously and synchronously the memory image that is the field that presents the concrete value of variables, and the target domain world that is the field that presents logically the data structures processed by the program. Learners can observe and compare program code, memory image, and target domain world by using TEDViT; hence, it is expected to bring positive effects on learners’ understanding of pointers. Moreover, TEDViT visualizes the status of the target domain world according to the visualization policy defined by the teacher in order to allow teachers to set their instruction content based on the growing variety of learner background knowledge. Therefore, it is also expected to support teachers’ instructions and class managements appropriately.

In this paper, we describe a classroom practice for software engineers aimed at allowing them to cultivate a better understanding of pointers in connection with memory models. For that aim, the practiced class includes learning activities that each participant individually observes and compares program code, memory image, and target domain world. Following the description of TEDViT in the next section, we describe the classroom practice and the questionnaires conducted after the practice. Based on the analysis of the answers, we discuss that learning based on observations and comparisons of three world visualizations would have a certain effect to understand pointers and that introducing TEDViT into classrooms could contribute to teachers’ efficient and effective class management.

## TEDViT: Teacher Explaining Design Visualization Tool

We focused on TEDViT as the program visualization system introducing classroom for understanding pointers. TEDViT has two characteristic features that are different from existing systems. First, TEDViT can visualize the data structures processed by target programs in two ways: memory image of variables and status of the target domain world. Second, TEDViT can visualize the target domain world according to visualization policy defined by a teacher. In this section, we describe these two features of TEDViT and overview of related existing systems.

### Visualizing memory image of variables and status of target domain world

**Fig. 1:**
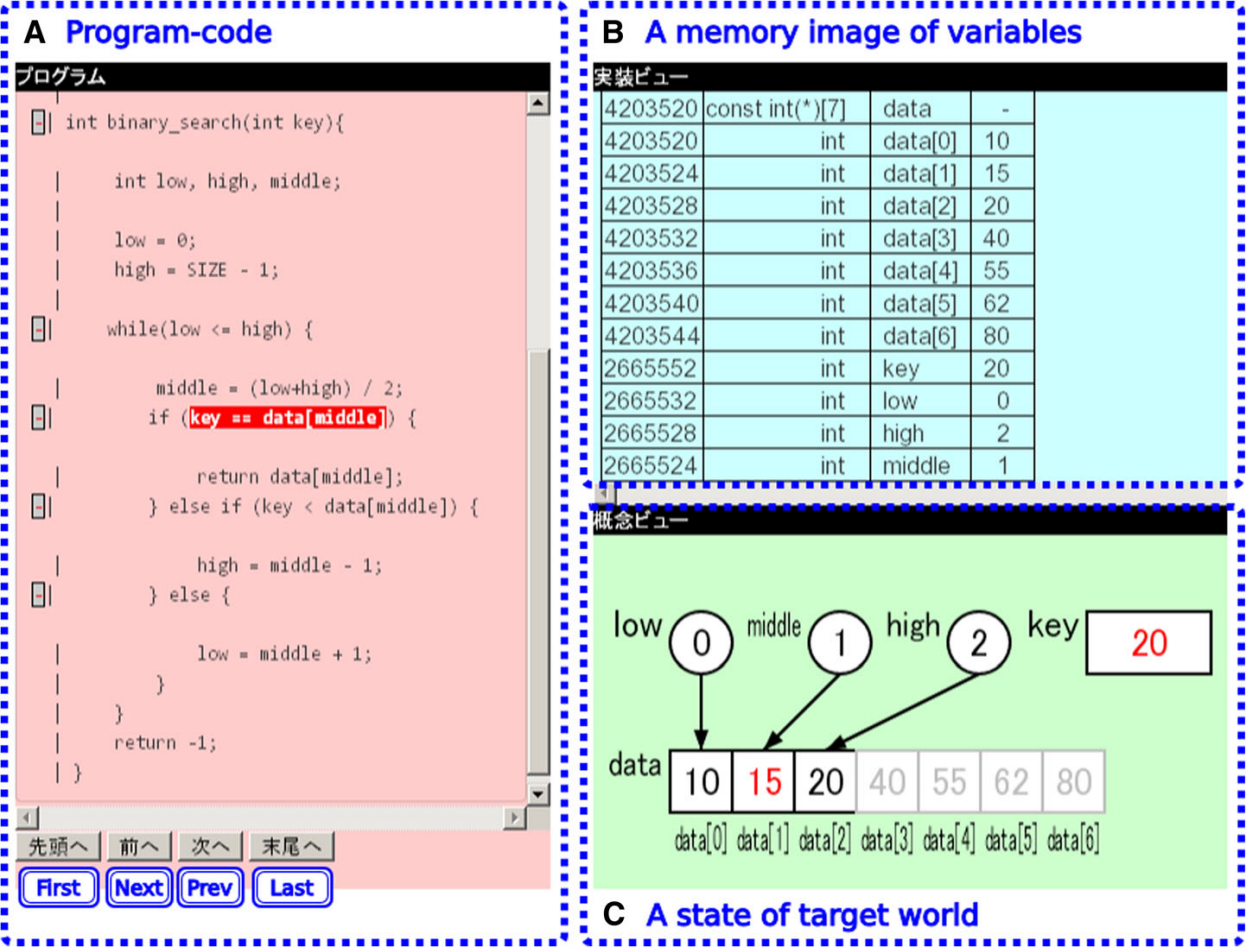
Overview of learning environment generated by TEDViT, including three visualization fields (**a**), (**b**), and (**c**)

TEDViT visualizes the data structures processed by a target program in two ways, memory image of variables and status of target domain world, and provides a learning environment where learners can observe and compare both visualizations. Figure 1 shows a learning environment generated by TEDViT. The environment consists of three fields: the data structures processed by the program in (A) are visualized in the two fields in (B) and (C). TEDViT reproduces a series of memory images of variables in (B) for each step of the program’s execution with table format. Each record includes the following attributes: address of variable on memory, variable type, variable name, and concrete value of variable. TEDViT also reproduces a series of statuses of the target domain world in (C) that visualizes logical data structures. We will explain the visualizations in (C) in the next subsection. When a learner clicks the “Next” or “Prev” button, the highlight in (A) moves to the next or previous statement in the program code; the memory image in (B) is updated according to the values of the variables after executing the highlighted statement; and the corresponding status of the target domain world is visualized in (C). TEDViT simulates statement execution step by step so that the learner can understand the program’s behavior by observing the changes of the target domain world in (C). Simultaneously, the learner can understand the concrete memory image in each execution step and the concrete expression of the data structures by observing and comparing the world in (C) with the memory image in (B).

The current version of TEDViT has the following features in visualization of the memory image:

- Visualizing the variable size and the size of the data pointed by the variable if it is a pointer.
- Visualizing the memory table where the row height corresponds to the variable size.
- Highlighting the operand variable in the address operation if the focusing statement includes an address operation.

**Fig. 2:**
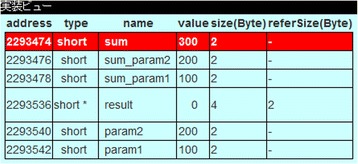
Example of memory image visualization in our practice

Figure 2 provides an example of memory image visualization practically used in our practiced classroom. Here, the focusing statement includes the address operation with operand variable *sum*.

### Visualizing status of target domain world based on teacher’s intention

For example, a teacher might draw an object in a horizontal layout when the instruction target is to sort an array, whereas the teacher might draw the array in a vertical layout for a stack. Changes in visualization policy such as this are derived by fitting the instruction content to the learners’ background knowledge. For example, if the learners sufficiently understand a stack, drawing either object in a horizontal or vertical layout would be acceptable to the learners. Similarly, the teacher would not need to draw the temporary variable in a task that swaps the values of two variables for non-novice learners.

A typical method for providing visualized data structures to learners is to show slides and/or movies made with presentation and video editing software. Nevertheless, these materials cannot be used for certain learning activities, such as learners observing program behavior where input data are changed individually, because the input data are fixed. The other method to do with allowing learners to change input data is to provide target program to learners, including graphic drawing functions. However, this is also not realistic because it might burden teachers with troublesome coding, such as creating, updating, and deleting drawing objects with name resolution that involves name spaces, scopes, and so on.

**Fig. 3:**
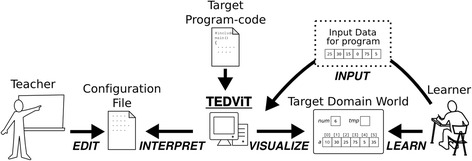
Relationship among teacher, learner, and TEDViT (Yamashita et al., 2016)

To resolve this problem, a function has been implemented for teachers to define the policy for drawing a status of the target domain world according to their own intent. The teachers can create or edit a configuration file independently from the target program file. TEDViT interprets such visualization policy by scanning the configuration file and visualizes the target domain world according to it. The learners can then observe the program behavior in the target world visualized in accordance with the teacher’s intent. The relationship among teacher, learner, and TEDViT is shown in Fig. 3.

### Related works

The concept to support learners’ understanding by visualizing data structures and their behavior along with the statement executions of target program in certain ways has relatively long history in programming education research. Thus far, several program visualization systems have been developed. The reason of our adoption of TEDViT among these existing systems is that we have dissatisfaction with many of the existing systems as follows:

- The visualization provided by existing systems is too much abstracted to learn the target closely related to hardware, such as pointers.
- If the abstractions of data structures by existing systems were differ from teacher’s explanations in the classrooms, learners may confuse in understanding by providing various visualization objects each of which has a different abstraction.
- Using systems that allow teachers to arbitrarily set the visualization fields observed by learners, it takes considerable time to complete visualization settings.

Many existing systems such as Jeliot 3 (Moreno, Myller, Sutinen, & Ben-Ari, 2004; Ben-Ari et al., 2011), NoobLab (Neve, Hunter, Livingstone, & Orwell, 2012), and LEPA (Yamashita et al., 2016) reproduce the behaviors of programs by visualizing logical data structures processed by target program and their changes made by statement execution. However, these visualizations involve certain abstractions of data structures which are established independently in each system. These abstractions often conceal detailed data which teachers might want to make students observe in a learning target closely related to hardware, such as pointers. For example, iList (Fossati, Eugenio, Brown, & Ohlsson, 2008) visualizes logical data structures targeting linked lists and supports learners to understand algorithm behavior and role of code statement, allowing learners to operate the visualized structure by inputting code fragments. The concrete values of pointer variables are concealed in iList, and hence, the way in which each visualized object is located on memory is not made explicit. Depending on the learning target, teachers are highly likely to explain program behaviors based on memory models. However, almost all of these existing systems do not have a function that allows teachers to alter the abstraction of visualizations.

Moreover, in algorithm visualization systems such as TRAKRA 2 (Malmi et al., 2004), the scope of abstraction is extended to program codes. TRAKRA 2 reproduces algorithm behaviors by learner’s GUI manipulations on visualized logical data structures, and hence, it is expected to have an effect to understand algorithms. However, the visualization of TRAKRA 2 often cannot be immediately expressed by some lexical items and syntactic fragments provided by programming language. If learners reached an appropriate level of understanding of an algorithm, they would have to write a code with combining various syntactic fragments complicatedly, such as self-referential data structures. In programming education, the gap between an algorithm and its implementation method needs to be bridged by a certain way. In the learning target such as pointers, the system introduced into the class is required to deal with the visualizations of not only the statuses of target domain world with a certain abstraction but also the less abstracted statuses of notional machine and actual program codes controlling it (Sorva, 2013).

Also, it would be an obstacle to introduce these systems into the class that visualizations of program behaviors have fixed policies established beforehand and independently by the system’s developer team. In introducing these systems into the class, the teachers would have to accommodate their explanation to the visualization policy established by each system. Because teachers would avoid students’ confusion in understanding by providing various visualization objects, each of which has a different policy. It would be a burden for teachers designing classrooms, and hence, it would be one of the factors that teachers cancel their introduction of program visualization systems. As the range of educational opportunities for introducing students to programming has been expanded, we consider that systems introduced into the class need a function that enables teachers to adjust the visualization policies. For example, Gries and Gries (2002) proposed the visualization method of memory model for teaching Java and object orientation to novice learners. The system introduced into the class should deal with visualizations reflecting teacher’s intention, as much as possible.

We adopted TEDViT as the system introduced into the class because it satisfies these requirements. TEDViT visualizes program codes, memory images, and logical data structures, simultaneously and synchronously, and allows teachers to define visualization policy of logical data structures. Other systems capable of arbitrary visualization definitions include ANIMAL (Rössling & Freisleben, 2002); however, the cost of visualization definitions in ANIMAL tends to be relatively high. By using ANIMAL, teachers can define arbitrary policy with a script language named AnimalScript and can provide arbitrary visualization to their students. Although the description capability increases significantly by using the script language, the cost associated with learning the language is a matter that cannot be ignored. Moreover, the sizes of script codes also tend to be large relatively. For example, the sample script for a bubble sort algorithm bundled in ANIMAL consists of 170 lines of code. Comparing it with the size of source code for bubble sort, it is hard to say that the script size is sufficiently small.

Visualization policies in TEDViT is defined by a set of drawing rules in CSV format, and any definition could be completed in practical time with some experience in rule definitions. The times required to complete rule definitions are approximately the same as slide creations with some presentation software. However, the teaching material created through the visualization definitions in TEDViT would be more useful because TEDViT can reproduce the program behavior without rule modifications, even if the target data processed by the program change. Furthermore, development of graphical interface for visualization definition in TEDViT has proceeded to reducing the cost (Tezuka et al., 2016).

## Methods: Classroom practice for understanding pointers

In this section, we describe the classroom practice that used TEDViT for understanding pointers. The practice was conducted along with a scenario that included activities for observing and comparing memory images and the target domain world. It was incorporated into four sessions in open lecture for software engineers. Because the participants in the practice were not students but engineers already employed, we evaluated the effect of the practice based on questionnaire survey conducted after the practice. Hamamatsu Embedded Programming Technology Consortium (HEPT) is a collaborative consortium with industries and the university to which some of the authors belong; HEPT is also training system engineers. HEPT offers a training course for software design and development engineering, including lectures on programming in C. Our classroom practice was incorporated into four sessions of these lectures and conducted at 13 May 2015. The practice participants were 15 software engineers responsible for software development, but without much experience in C programming, as listed in Tables 1 and 2.

**Table 1:** Number of participants with different years of programming experience

Years of experience	Less than 1 year	1–3 years	3–5 years	5–7 years
Number of participants	5	7	1	2

**Table 2:** Number of participants with experience in each language (multiple replies allowed)

Programming language	C	C++	C#	Visual Basic	Java	Perl
Number of participants	13	6	3	4	4	1

The goal of the sessions is to understand how to design and implement a function that can take arbitrary data types using function and generic pointers. In order to achieve this goal, the learners need to understand “relationships among types, variables, and pointers,” “pointers in function arguments,” “pointers as return values,” “pointer operations,” and “relationships between pointers and arrays” in connection with a memory model. Pointer is a mechanism specific to a few programming languages such as C/C++. It is difficult for engineers who usually use other language such as Java, VB, and Perl to understand these learning targets, even if they had sufficient experience in software development. The sessions into which our practice was incorporated were held to support those engineers to understand pointers.

The practiced sessions and the learning sections in each session are the following:

1. Variables and pointers
  - Pointer operators and memory model
2. Functions and pointers
  - Calls by value
  - Calls by pointer
  - Return values with pointers
3. Arrays and pointers
  - Relationship between array names and pointers
  - Relationship between arithmetic operations on pointers and array index operations
  - Memory model of multidimensional arrays
  - Handling an array to reverse the order
4. Generic pointers
  - Operations on generic pointers
  - Sample library of integer stacks

Aiming to the goal achievement, we introduced TEDViT into the session classes and incorporated learning activities to observe statuses of the target domain world associating with statuses of the memory image into the session learnings. We expected that these activities could contribute to cultivating better understanding of pointers. In the practice, four sessions were conducted in above order by 90 min each. Each learning section consisted of receiving the classroom lecture, observing the program behavior with TEDViT, practicing with a coding exercise, and receiving an explanation of the solutions for the exercise. We planned to allow the participants to use TEDViT mainly for observing program behavior so that they could observe and compare the memory image of variables and the target domain world by operating individually the learning environment visualized by TEDViT. We also observed that the teacher of the session classes used TEDViT to explain the point of an exercise solution with connections between the status of the target domain world and the memory image of variables.

**Fig. 4:**
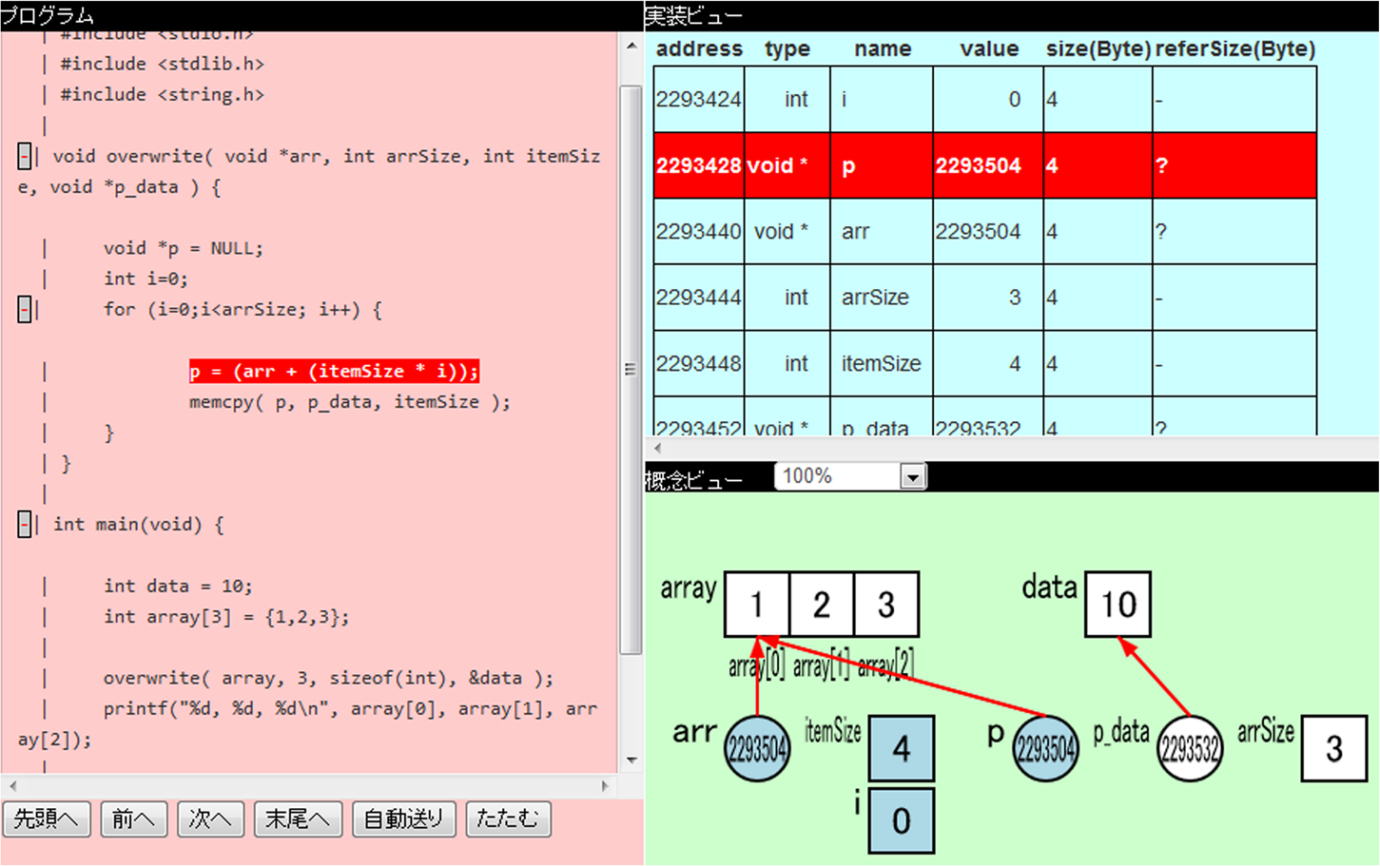
Example of environment for learning generic pointers

The teacher prepared the target program codes observed by the participants with TEDViT in the practice and the configuration files for the visualization of the target domain world. The number of prepared teaching materials (i.e., program codes and configuration files) was ten. The visualizations defined by the teacher in accordance with the teacher’s own intent included arrow objects for representing the relationships between pointers and pointed variables, and coloring to highlight the objects on which the teacher intended the participants to focus. Figure 4 provides an example of the actual learning environment in the practice.

### Results: Classroom practice evaluation

It was difficult to conduct a certain test for the evaluation of the learning effect with TEDViT because the participants in the practice were not students, but engineers already employed. For the same reason, participants’ achievement codes in coding exercises were not assessed. Hence, our evaluation of the classroom practice is based on an analysis of the answers of two questionnaires conducted after the practice. One of them (Q1) was about learning with TEDViT, intended to evaluate how much the participants were satisfied with learning supports of pointers by TEDViT. The other (Q2) was about the learning content in the session, which is the same as the questionnaires conducted every past year, intended to evaluate the effect of the practiced sessions with TEDViT comparing corresponding past sessions without it.

The questionnaire Q1 contains six items.

Q1-1. Asks how much TEDViT contributed to understanding C programming using five-point scale.
Q1-2. Asks how much TEDViT contributed to ascertaining wanted matters using five-point scale.
Q1-3. Asks how much TEDViT was needed for learning using five-point scale.
Q1-4. Asks whether the teaching materials are regarded as useful from among the prepared ten materials (allows multiple answers).
Q1-5. Asks for comments on the advantages and disadvantages of learning with TEDViT.
Q1-6. Asks for comments on the insufficient or inconvenient functions of TEDViT.

Table 3 provides an average of the answer scores and standard deviation as dispersion indicator of the answer scores for Q1-1, Q1-2, and Q1-3. These scores suggest that the participants were basically satisfied with the learning support provided by TEDViT. Table 4 lists the teaching materials found useful by participants (Q1-4). These results suggest that participants of the practice were generally satisfied with learning support by TEDViT.

**Table 3:** Average and standard deviation of answered scores in Q1-1, Q1-2, and Q1-3

	Average	SD
Q1-1	4.60	0.49
Q1-2	4.40	0.61
Q1-3	4.33	0.60

**Table 4:** Number of answers indicating useful teaching materials in Q1-4 (multiple replies allowed)

Teaching material	*N*
Pointer operators and memory model	7
Calls by value	3
Calls by pointer	7
Return values with pointers	4
Relationship between array names and pointers	3
Relationship between arithmetic operations on pointers and array index operations	3
Memory model of multidimensional arrays	4
Handling an array to reverse the order	5
Operations on generic pointers	9
Sample library of integer stacks	9

The questionnaire Q2 has five items, all of which are same as those conducted over several years at the HEPT training course:

Q2-1. Asks how much the participant was interested in learning the session content using 5-point scale.
Q2-2. Asks how well the participant understood the session learning content using 5-point scale.
Q2-3. Asks how fast the participant perceived was the teacher’s progress in the session using 5-point scale (1 = too slow, 5 = too fast).
Q2-4. Asks how difficult the session learning content was using 5-point scale (1 = too easy, 5 = too difficult).
Q2-5. Asks how difficult the exercises were in the session using 5-point scale (similar to Q2-4).

Table 5 provides the average and standard deviation of each answer score in Q2, including those in the questionnaire conducted in 2013 and 2014. The corresponding learning sessions in 2013 and 2014 were conducted by the same teacher as those in 2015 without TEDViT. The sessions in 2013 were held at 5 June 2013 for 30 engineers, and those in 2014 were held at 28 May 2014 for 16 engineers. The answer scores of sessions in 2015 suggest that participants were generally satisfied with practiced sessions with TEDViT.

**Table 5:** Average and standard deviation of answered scores in Q2 including past surveys

	2015 (TEDViT)		2014		2013	
	Average	SD	Average	SD	Average	SD
Q2-1	4.67	0.60	4.31	0.58	4.33	0.91
Q2-2	4.07	0.77	4.38	0.93	3.17	1.10
Q2-3	3.07	0.44	3.06	0.66	3.37	0.55
Q2-4	3.27	0.57	3.13	0.86	3.53	0.76
Q2-5	3.47	0.72	3.25	0.83	3.60	0.66

## Discussion

Our practice described in the previous section was conducted not in virtual classroom for experiment, but in the actual open lecture. Hence, we need to take into account that the knowledge obtained from the practice might have some limitations, though it would be more practical. The limitations of our practice are that we could not procure an enough number of participants to measure the learning effects and could not conduct a certain test for the evaluation of the effects. In this section, we discuss that the learning supports provided by TEDViT were accepted positively by the participants and that TEDViT could support teacher’s class management by reducing unevenness among learners in understanding.

According to the results in Table 3, the participants of our practice rated highly the contributions of TEDViT to understanding C programming. In the open lecture in which our practice was conducted, the participants’ background knowledge tend to be extremely uneven. The positive results regardless of this tendency suggest that participants strongly support learning activities based on observing and comparing program code, memory image, and target domain world. Moreover, according to the results in Table 4, the indicated teaching materials which were answered by more than five participants are divided broadly into the following two categories:

1. Learning items with complicated behavior, such as “handling an array to reverse the order” and “sample library of integer stacks”
2. Learning items where a learner is required to grasp the relationship between the target domain world and memory model, such as “pointer operators and memory model,” “calls by pointer,” and “operations on generic pointers”

We consider that the participants who felt to be supported by the visualized target domain world to understand the program code with complicated behavior would prefer materials in the former categories. This suggests that the visualization defined by the teacher successfully supports participants to understand program behavior. On the other hand, the positive answers to the latter categories suggest that not a few participants felt to be supported to grasp the relationship between the memory image and the target domain world by observing and comparing the corresponding fields visualized by TEDViT. This suggests that synchronous and simultaneous visualization of program code, memory image, and target domain world contributes to supporting learners to understand the relationship.

**Fig. 5:**
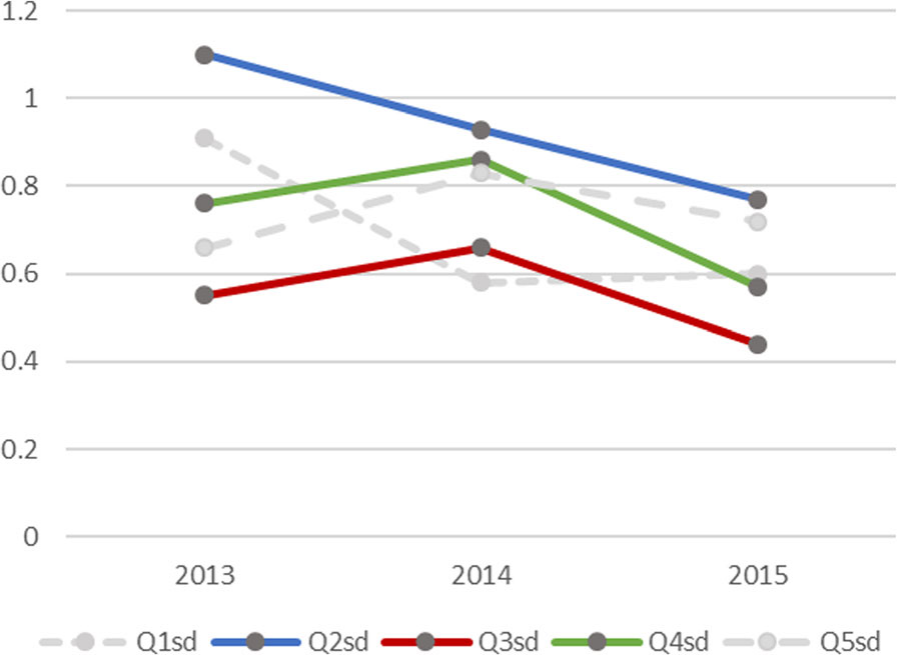
Transitions of the standard deviations of answered score in Q2

According to the results in Table 5, practiced sessions with TEDViT would contribute to participants’ understanding in the same degree as sessions without TEDViT. This means that TEDViT did not impede the participants’ learnings in the classroom practice. We can also find that the standard deviations of answer scores on understanding degree (Q2-2), session speed (Q2-3), and session difficulty (Q2-4) in 2015 are lower than those in 2013 and 2014. Figure 5 provides transition of standard deviation by means of a graph. These reductions of standard deviations mean reduction of unevenness in levels of understanding of the session contents and in levels of difficulties of following class among participants.

We conducted an *F* test to evaluate the variance uniformity of answer distributions on each items of Q2 for two groups: one is the answer in 2015 and the other is the answer in 2013 and 2014. Table 6 provides the result of the *F* test. Table 7 shows the result of the *t* test conducted for the same two groups as *F* test. Assuming significance level of 0.05, statistically significant differences were not found through the both test. However, there were no participants of sessions in 2015 who answered “not understandable at all” or “slightly not understandable” in Q2-2, in contrast to the sessions in 2013 and 2014, where some participants did provide these answers. Moreover, we conducted an interview with the teacher of practiced classes and received a comment that unevenness among participants in progress in their exercises could be reduced by TEDViT, and hence, he felt easy to teach the practiced classes. These suggest that individual learning support successfully reached every participant by using TEDViT.

**Table 6:** The result of *F* test for Q2 answers in 2015 versus those in 2013 and 2014

	*F*(14, 46)	*p*
Q2-1	0.55055	0.2234
Q2-2	0.44820	0.1034
Q2-3	0.56908	0.2503
Q2-4	0.52110	0.1838
Q2-5	0.98862	0.9594

**Table 7:** The result of *t* test for Q2 answers in 2015 versus those in 2013 and 2014

	*t*	*df*	*p*
Q2-1	1.8412	31.695	0.0750
Q2-2	1.8238	35.550	0.0766
Q2-3	− 1.2777	31.126	0.2108
Q2-4	− 0.5976	32.670	0.5542
Q2-5	− 0.0064	23.745	0.9949

As mentioned in the “Related works” section, several program visualization systems have been developed so far, and several positive effects have been reported for learning with those systems. Nevertheless, there has been very few works that focused on reducing unevenness among learners in understanding. Rather, learning support systems are evaluated on the premise of the unevenness based on adaptive learning. For example, Brusilovsky and Spring (2004) developed a system called WADEIn, which has a function that visualizes natural language explanations adaptively depending on learners’ understanding. They introduced WADEIn into pointer learning and obtained positive evaluations from more than 80% subjects. However, teachers may hesitate to introduce a system like WADEIn into actual classrooms, because adaptive visualizations will take a high cost of preparation for a wide variety of understanding among learners and it will be difficult to plan class schedules assuming progress in learners’ exercises with adaptive system. The problem of preparation cost and scheduling difficulty is more serious especially in open lectures where teachers cannot assume levels of learners’ background knowledge. We consider that our practice is regarded as one of the instances to address the unevenness among learners in understanding with reducing the preparation costs. The comments by the teacher of our practice were positive in introducing TEDViT, and we conclude that our classroom practice could be conducted successfully by introducing TEDViT.

Based on these discussions, we can summarize knowledge obtained through the classroom practice as follows:

- Learning based on observations and comparisons of target program code, memory image, and target domain world would contribute to understanding of pointers.
- TEDViT could contribute to reducing unevenness among learners in progress in their exercises and could support teacher’s class management.

The limitation of our practice is that we could not procure an enough number of participants for the practice, because our practice was incorporated into the sessions of an open lecture for engineers already employed. However, we believe that continuous practice will suppress this matter. We plan to continue the classroom practices of programming education introducing TEDViT. Other limitations include that our evaluation of learning effect with TEDViT was based only on the answer scores in questionnaires. It was difficult to conduct a certain overall test for the evaluation of the learning effect with TEDViT because the participants in the practice were not students, but engineers already employed. We plan to develop some way of direct evaluation realizing lower externalization cost of participant’s learning achievement and to examine some indirect evaluation such as recording and analyzing participant’s operation logs of TEDViT.

## Conclusion

In this paper, we described a classroom practice for understanding pointers and a program visualization system introduced into the practice. Pointer is one of most difficult learning targets for novice learners to understand as well as recursion. Introducing program visualization systems to programming classes is expected to support novice learners to overcome these difficulties. Program visualization systems visualize the data structures processed by the target programs in certain ways and support learners in understanding the programs and algorithms by making their behavior visible. However, concrete values of pointer variables are often concealed in such visualizations, and hence, the way in which each visualized object is located on the memory is not made explicit. Therefore, novice learners often fail to grasp the relationship between program behavior and program code and reach a learning impasse.

To address this issue, we focused on TEDViT, a system providing an environment to observe and compare program code, memory image, and target domain world. TEDViT visualizes three visualization fields simultaneously and synchronously and provides a learning environment where learners can observe and compare the three, that is, the target program code, the memory image representing the concrete values of the variables, and the target domain world representing logically the data structures processed by the target program. Moreover, in order to allow teachers to set their instruction content based on the growing variety of learner background knowledge, TEDViT visualizes the status of the target domain world according to the visualization policy defined by the teacher. Using TEDViT, teachers can provide flexible visualizations that are consistent with the teacher’s class instructions to the learners, and learners can be less confused with the visualizations.

We conducted a classroom practice introducing TEDViT for understanding pointers in connection with a memory model. The participants in this practice were not university students, but software engineers responsible for software development. They learned program behavior by observing and comparing the memory image and target domain world visualized in the learning environment of TEDViT. In the questionnaire conducted after our practice, we obtained generally satisfactory answers for TEDViT. The analysis of answered scores suggested that TEDViT could contribute to reducing unevenness among participants in levels of understanding and that individual learning support successfully reached every participant by using TEDViT. In the questionnaire, some participants commented that the observations of the memory image in learning pointers were especially valuable. The comments by the teacher of our practice were also positive in that unevenness among participants in progress in their exercises could be reduced by TEDViT, and hence, he felt easy to teach the practiced classes.

These results suggest that synchronous and simultaneous visualization of program code, memory image, and target domain world contributes to understanding of pointers. Although we need to consider that a statistically insufficient number of the participants of our practice may influence the reliability of evaluation results, continuous practice would suppress this matter. We can conclude that the framework of our classroom practice could be conducted successfully by introducing TEDViT. In future work, we plan to conduct more educational practices and evaluate the effectiveness of using TEDViT with higher reliability.
